# Crystal Memory near Discontinuous Triacylglycerol Phase Transitions: Models, Metastable Regimes, and Critical Points

**DOI:** 10.3390/molecules25235631

**Published:** 2020-11-30

**Authors:** David A. Pink, Marjorie Ladd-Parada, Alejandro G. Marangoni, Gianfranco Mazzanti

**Affiliations:** 1 Physics Department, St. Francis Xavier University, Antigonish, NS B2G 2W5, Canada; 2 Food Science Department, University of Guelph, Guelph, ON N1G 2W1, Canada; amarango@uoguelph.ca; 3 Department of Physics, Stockholm University, 114 19 Stockholm, Sweden; marjorie.ladd-parada@fysik.su.se; 4 Department of Process Engineering and Applied Science, Dalhousie University, Halifax, NS B3H 4R2, Canada; gianfranco.mazzanti@dal.ca

**Keywords:** triacylglycerol molecules, discontinuous phase transition, crystal memory, mathematical model, superheated regime, experimental data, modified Vogel-Fulcher-Tammann equation, critical point, crossover regime

## Abstract

It is proposed that “crystal memory”, observed in a discontinuous solid-liquid phase transition of saturated triacylglycerol (TAG) molecules, is due to the coexistence of solid TAG crystalline phases and a liquid TAG phase, in a superheated metastable regime. Such a coexistence has been detected. Solid crystals can act as heterogeneous nuclei onto which molecules can condense as the temperature is lowered. We outlined a mathematical model, with a single phase transition, that shows how the time-temperature observations can be explained, makes predictions, and relates them to recent experimental data. A modified Vogel-Fulcher-Tammann (VFT) equation is used to predict time-temperature relations for the observation of “crystal memory” and to show boundaries beyond which “crystal memory” is not observed. A plot of the lifetime of a metastable state versus temperature, using the modified VFT equation, agrees with recent time-temperature data. The model can be falsified through its predictions: the model possesses a critical point and we outline a procedure describing how it could be observed by changing the hydrocarbon chain length. We make predictions about how thermodynamic functions will change as the critical point is reached and as the system enters a crossover regime. The model predicts that the phenomenon of “crystal memory” will not be observed unless the system is cooled from a superheated metastable regime associated with a discontinuous phase transition.

## 1. Introduction

In recent years, the concept of “Crystal Memory” has been extended from describing polymer thermodynamics near phase transitions to describing the seeming ability of a triacylglycerol (TAG) melt to “remember” the solid structure from which it melted via a discontinuous (first-order) phase transition [1,2,3,4,5]. Discontinuous phase transitions of single-component systems exhibit regimes of metastability, something which continuous (second-order) phase transitions do not. It is sometimes not appreciated that metastable regimes arise, not only for supercooling, but also for superheating. It is known, for example, that a superheated regime exists not only for polymers [6] but also for smaller molecules such as glucose and fructose [7] and sucrose [8].

Accordingly, we expect that a superheated regime would be observed for TAG heating. One characteristic of metastable regimes is the coexistence of the two phases which characterize the system, for temperatures below and above the phase transition temperature. The cases considered here are the solid and liquid states of saturated TAG systems. A study to map the limits of this phenomenon in TAG systems was undertaken by Wang [1].

In describing their experimental protocols, many authors write statements to the effect that their samples of TAG systems, were heated at a specified temperature [9] order to “erase crystal memory” without giving a reason as to why that temperature is sufficient. This is related to the basic question of what is “crystal memory” near TAG crystal phase transitions. This is a phenomenon, reported by many ([1,9] and references therein) and so it is a fair question to ask what are the physical reasons for its existence, and what is the process by which it arises?

This phenomenon has been studied for linear polymers [10,11,12], as well as for cocoa butter [13] and fatty acids [14]. Those papers addressed questions regarding the role played by polymer self- and mutual-entanglement in the kinetics of memory effects.Recently, TAG crystallization has been studied [15,16,17,18]. Coexistence of solid and liquid state TAGs were reported [16]. The second paper [11] is especially interesting because it showed that the power law behavior of the overall crystal growth rate, found previously to be different for melts with and without memory, and interpreted in terms of changes in the structure and/or the dynamics of the entangled melt states, was not found in their NMR results. These results for the power law behaviour were found to be identical for melts with and without memory, therefore excluding any large effect of the “memory” on melt structure or dynamics. This supported conventional interpretations in terms of persisting nuclei. Muthukumar [19] developed a theory which assumed the existence of an intermediate inhomogeneous melt state which might be related to polymer entanglement.

The intent of this paper is to propose that “crystal memory” in cases of TAG systems is a consequence of the well-known phenomenon of metastability associated with discontinuous (i.e., first-order) phase transitions, and in which components of two equilibrium phases can coexist at a given temperature and pressure.

Although many studies of TAG metastability are concerned with supercooled systems (e.g., Sato and Ueno [20]), the “crystal memory” effect is reported for the superheated regime which also exhibits metastability. We denote the equilibrium phase transition temperature by $T^{*}$. By “supercooled” and “superheated”, we mean temperatures, in the neighbourhood of $T^{*}$, below and above the discontinuous phase transition temperature, $T^{*}$.

In what follows, we are not concerned with any stresses set up inside the crystalline fats. Stress tensors can be defined for a solid, (e.g., Baidakov [21]) but here we do not admit the high hydrostatic pressure or deformations which would require the consideration of stresses. Much work has been done on modelling TAGs using atomic scale molecular dynamics [22,23,24] though they did not attempt to simulate metastable phases. Ionov et al. [25], however, addressed questions regarding the identification of metastable regimes from molecular dynamics simulations.

We shall make use of the results of Pink et al. [26]. The intent of that paper was to show that an a priori plausible model could obtain a transition enthalpy in agreement with the wide range of values from experiment, and successfully predict Raman spectra intensities through a discontinuous phase transition. The fact that it predicted metastable regimes extending over tens of degrees was of no relevance to that paper. It is here that we make use of that result.

On the whole, classical thermodynamics or larger-scale mean field models in which correlations are essentially ignored (e.g., the Avrami model) are the approaches of choice for most models used in food science. These are mesoscale or macroscopic approaches. An alternative approach is to begin with models of the molecules involved and utilize statistical mechanics. This approach enables one to relate models of the molecular structure of a system to its large-scale thermodynamic properties [27]. The observability of metastable states depends upon their lifetime, i.e., their kinetics, in that regime. Although superheated metastability has been studied, most experimental studies of TAGs have involved the supercooled (i.e., below the phase transition temperature) metastable regime (e.g., Mykhaylyk et al. [28] and Sato and Ueno [20]). One advantage of the classical thermodynamic approach or the use of larger-scale models is that someone else has done the work in setting up the equations, and thought must be put in to interpreting the output. The advantage of the use of statistical mechanics applied to molecular models is that, in principle, one can predict measurable macroscopic-scale phenomena from calculations involving molecular scales. Questions regarding the molecular interactions (dispersion and short-range repulsive interactions, electrostatics) to be used, are frequently easier to answer than those regarding reaction rates or free energies. One point that is frequently unappreciated is this: a model used only to “explain” experimental data cannot be falsified. Any model used must be able to predict new phenomena, or at least outline how it can be tested and falsified.

We have chosen to create molecular-scale models and to use the methods of statistical mechanics to understand the various phase transitions and changes that one can obtain. In the next section, we outline a model of a very simple TAG phase transition, followed by a suggested explanation of the phenomenon of “crystal memory” as a manifestation of metastable states. This model is, however, particularly simple in that it exhibits only a single discontinuous phase transition. Since TAGs can have at least three phase transitions between solid α, β’, and β phases and liquid oil phases, the single-transition model used here cannot account for subtleties in the phase transitions. However, we shall show that some characteristics of “crystal memory” can be understood by this model, and that we are able to make testable predictions. We accompany this with a brief summary of portions of recent extensive work by Wang [1] who explored and described the boundaries and characteristics for cases of single- and multi-component TAG systems. Here, we shall be concerned only with single-component saturated TAG systems. Finally we attempt to make predictions which arise from the model. Although we expect that the model has a wider application, we make predictions that depend upon certain limits which we know to be true for saturated TAG molecules.

## 2. Results

In this section, we describe the results to understand the shape of the experimental holding time-temperature data by showing how they are related to the properties of the metastable regime of our theoretical model. The experimental process establishes the boundaries of the region in which “crystal memory” is observed to occur and the region in which it does not occur. To understand our results and how they account for the experimental observations, it is necessary to describe the model, how the theoretical time-temperature relation is obtained from a modified Vogel-Fulcher-Tammann equation, and the procedure for acquiring the experimental data.

### 2.1. Theory and Models: The g-e Model

**Figure 1:**
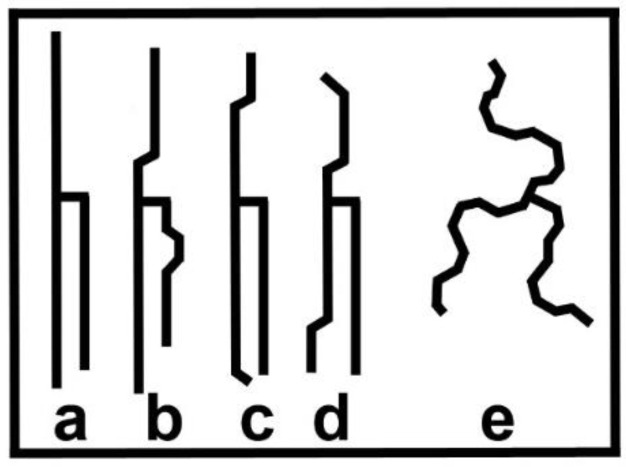
Pictorial simplification of the saturated alkyl chains of a triacylglycerol (TAG). **a**–**d**: Possible ground, or low-energy, state conformations. **e**: Possible conformation in the excited state. Many effectively-degenerate conformations are feasible.

In order to study whether the saturated TAG solid-liquid phase transition as described by Larsson [29] and Cebula et al. [30] was in accord with experiment, a statistical mechanical model of a saturated hydrocarbon chain solid-liquid phase transition was devised by Pink et al. [31] and its predictions compared to measurements of transition enthalpies and the temperature-dependence of Raman spectra. Here, we shall use a modified form of this model. The model represented the hydrocarbon chains as two-dimensional structures which defined low-energy conformers, and its associated Raman intensities with each conformer. The effective Hamiltonian for the system of TAG molecules was:

(1) $$H=-½\underset{ij}{\sum }\underset{mn}{\sum }J_{mn}\left(i,j\right)P_{im}P_{jn}+\underset{i}{\sum }\underset{m}{\sum }\left[E_{m}-k_{B}T\ln\left(D_{m}\right)\right]P_{im}$$

where $J_{mn}\left(i,j\right)$ is the van der Waals interaction energy between two hydrocarbon chains, *i* and *j*, in conformational states *m* and *n,* respectively, $E_{m}$ is the energy of a single (isolated) chain in state *m*, and $P_{im}$ is a projection operator, equal to 1 when the chain labelled *i* is in state *m* and zero otherwise. A chain in state *m* possesses an effective degeneracy $D_{m}$, the number of states which possess energy $E_{m}$. In cases where there is a set of states possessing energies which are “close” compared to the energy differences with other sets of states, we might replace the set by a single effective state with a degeneracy equal to the number of states in that set. Part of a set of conformational states possessing similar low energies are shown in Figure 1a–d. These, together with others like them, could be combined into a single state which could dominate the thermodynamics at temperatures below a “chain-melting” phase transition temperature. The projection operators are used to identify states and to help in ensuring that no states are omitted in error when carrying out calculations.

It should be understood that H is not the Hamiltonian energy operator since it incorporates the degeneracies. However, it is convenient to use this operator to calculate the partition function without having to include the degeneracies in it, as well as utilizing it as the effective energy in order to perform Monte Carlo computer simulations (e.g., Pink et al. [31]).

The problem of calculating thermodynamic quantities was simplified by reducing the states of a given hydrocarbon chain to two states, yielding a “g-e model”: an effective, temperature-dependent ground state, *g*, composed of low-energy “*h*” conformers, and an effective excited state, *e*, comprising high-energy “*Y*” conformers [26]. A similar model was used by Doniach [32] to model fluctuations in phospholipid bilayers. The sum over *m* and *n* now run over the two states *g* and *e*. These states possess energies $E_{g}$ and $E_{e}$, and degeneracies $D_{g}$ and $D_{e}$. Some conformational states are illustrated in Figure 1. In order to make the model transparent, we mapped the projection operators, $P_{ig}$ and $P_{ie}$ onto the “pseudo-spin” operator, $\sigma_{i}$, and the unit operator, $I_{i}$, for the hydrocarbon chain labelled *i*.

Ignoring terms which are constant, the g-e model Hamiltonian-like operator is akin to that of an Ising model in a temperature-dependent “magnetic” field,

(2) $$H=-½\underset{ij}{\sum }J\left(i,j\right)\sigma_{i}\sigma_{j}-\underset{i}{\sum }H\left(i,j,\;T\right)\sigma_{i}$$

where $\sigma_{i}=P_{ig}-P_{ie}$, $I_{i}=P_{ig}+P_{ie}$, and $H\left(i,\;j,\;T\right)=H_{0}\left(i,\;j,\;T\right)-½k_{B}T\ln\left(D_{e}/D_{g}\right)$. The values of the pseudo-spin operator, $\sigma_{i}$, are +1 (“spin-up” representing state g) and −1 (“spin-down” representing state e). By mapping the projection operators onto pseudo-spin operators, we reduce this “g-e” model to the Ising model, thereby enabling us to make use of the great deal of work which has been done over many years on the Ising model. The interactions are taken to be obtained from London forces so that they are short-range. However, we include another interaction that is intended to represent the electrostatic interactions between glycerol groups of TAGs. Because of the short-range of the (attractive) dispersion interactions, we assume that only nearest neighbour interactions are important so that $J\left(i,j\right)\to J=\left[J_{gg}-2J_{ge}+J_{ee}\right]/4$ and $H_{0}\left(i,\;j,\;T\right)\to H_{0}=z\left[J_{gg}-J_{ee}\right]/4+½\left(E_{e}-E_{g}\right)$. This assumption is not necessarily true for cases involving electrostatic interactions. Nonetheless, even such a simple model as this suffices to make our case. If we assume that the electrostatic interactions (below) are attractive, then at non-zero temperatures, both $H_{0}$ and $k_{B}T\ln\left(D_{e}/D_{g}\right)$ are greater than zero. We obtain:

(3) $$H=-J/2\underset{\left(ij\right)}{\sum }\sigma_{i}\sigma_{j}-H\left(T\right)\underset{i}{\sum }\sigma_{i}$$

with $H\left(T\right)=H_{0}-½k_{B}T\ln\left(D_{e}/D_{g}\right)$ and where the notation $\left(ij\right)$ indicates that *i* and *j* are nearest neighbours.

### 2.2. Theory and Models: Some (Bulk) Thermodynamic Properties of the Model

**Figure 2:**
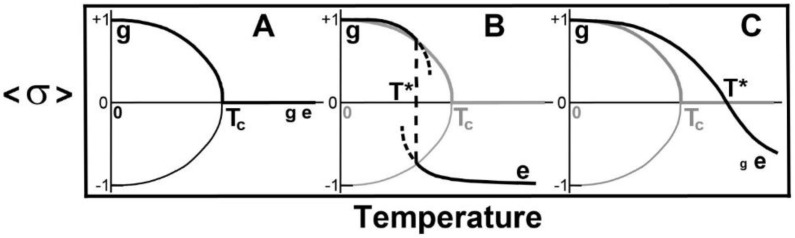
Schematic illustration of the temperature-dependence of $\langle \sigma_{j}\rangle \;=\;\langle \sigma \rangle$. Heavy lines show the solutions appropriate to the condition that, as T→0, $\langle \sigma \rangle \to 1$. **A**: Continuous transition for the field-free 2- and higher-dimensional Ising model. *T_c_* is the critical temperature of the continuous (second-order) transition. There is another solution, $\langle \sigma \rangle \to -1$, as T→0. We are free to choose which solution we want. With $H\left(T\right)$ present, a discontinuous (first-order) transition can arise. **B**: Discontinuous transition for T* < *T_c_*. Short dashed lines indicate the metastable regimes. **C**: T* > *T_c_*. The size of the letters *g* and *e* illustrate the probabilities of finding these states present at low and at high temperatures.

If the term in $H\left(T\right)$ is omitted and we employ statistical mechanics to obtain thermodynamic quantities, then, using analytical techniques or computer simulation, we obtain the solutions to the average value of $\sigma_{j}$, $\langle \sigma \rangle$, for the two- and higher-dimensional Ising model [33,34,35], and which is independent of *j.* The temperature-dependence is shown schematically in Figure 2A, with a continuous phase transition at temperature $T_{c}$. For the one-dimensional Ising model, $\langle \sigma \rangle \;=0$, for T>0. However, if we retain $H\left(T\right)$ then there are two limits: as T→0, $H\left(0\right)=H_{0}>0$ which favours the spin-up state, *g*, while as T→∞, $H\left(T\right)<0$ which favours the spin-down state, *e*. Accordingly, there exists a temperature, T*, at which $H\left(T^{*}\right)$ changes sign so that we obtain a “spin reorientation” transition, that is, a transition g↔e, when $H\left(T^{*}\right)=0$, where:

(4) $$T^{*}=\frac{2H_{0}}{k_{B}\ln\left(D_{e}/D_{g}\right)}$$

This g-e model exhibits a discontinuous phase transition when $T^{*}<T_{c}$ (Figure 2B) a continuous phase transition at the critical point when $T^{*}=T_{c}$, and a crossover when $T^{*}>T_{c}$ (Figure 2C). Experimentally, the transitions can exhibit seeming divergences or discontinuities (Figure 2B) or broad peaks (Figure 2C) in thermodynamic quantities.

### 2.3. Theory and Models. Mean Field Approximation

**Figure 3:**
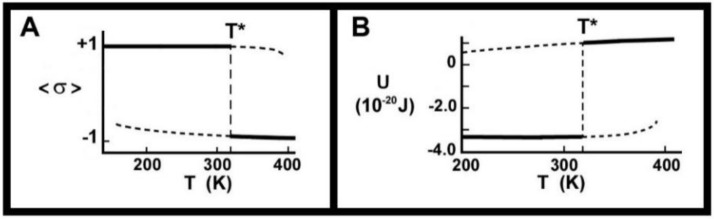
Order parameter, $\langle \sigma \rangle$, (**A**) and enthalpy, *U*, (**B**) as functions of temperature for trilaurin (LLL). The transition enthalpy, ∆U, is shown by the vertical dashed line at $T=T^{*}$. The four short-dashed lines show the regimes of metastability as calculated by a mean field approximation (modified with permission from Pink et al. [26]).

A mean field approximation [36,37] was used in the case of the Hamiltonian-like operator (Equation (2)) and thermodynamic quantities and Raman intensities were calculated [26] The mean field approximation yielded the well-known equation for $\langle \sigma \rangle$,

(5) $$\langle \sigma \rangle =\tanh\left[\left(K\langle \sigma \rangle +B\right)/k_{B}T\right]$$

where *K* is a function of the interaction energies and *B* a function of all the energies and the degeneracy term, $k_{B}T\ln\left(D_{e}/D_{g}\right)$. This requires a self-consistent solution for $\langle \sigma \rangle$, yielding both the stable and the metastable regimes, and which enables the calculation of the enthalpy, *U*. It was found that the most plausible *h-Y* model was also the only one to get close to the reported transition enthalpy, ∆U, of 81−124 kJ/mole [38]. This model reported a value of ∆U=86.7 kJ/mole for the TAG molecule trilaurin (LLL), with three identical saturated hydrocarbon chains. The calculated dependence of *U* upon *T* is shown in Figure 3.

One question that might arise is whether the mean field approximation will change the value of T*, since it is known to change the value of the critical temperature, *T_c_*, from the exact result in cases where that is known. The answer to the question is that, in this model, the value of $T^{*}$ is not changed by utilizing a mean field approximation. What can change, however, is the value of ∆U. Pink et al. [26] had not been concerned with the metastable regimes except to show that this model suggested that these regimes extended over at least 10 °C. The prediction of the temperature-dependence of Raman intensities characterizing extended chain states (Figure 1a−d) and the melted chain state (Figure 1e) was in good accord with the measured band ratios, but did suggest that the discontinuous phase transition might not be as abrupt as the model predicted [26]. An alternative explanation of the decrease in Raman intensity for $T<T^{*}$ might be that it is detecting polymorphic transitions. Despite its simplicity and possible defects, however, we returned to this model in order to use it for its prediction concerning metastable regimes. Work has been done on modelling metastable regimes [27,39].

### 2.4. Metastable Regimes

The existence of a discontinuous transition (Figure 2B) can give rise to metastable regimes corresponding to the short-dashed lines of Figure 3. These are superheated, ($T>T^{*}$), or supercooled, ($T<T^{*}$), regimes, for which the system is not in thermal equilibrium. The superheated regime, shown by the short-dashed line for $T>T^{*}$, can be accessed by heating the system from $T<T^{*}$ to $T>T^{*}$ sufficiently quickly so that the system moves along the lower short-dashed curve. For $T\approx T^{*}$ this regime will likely comprise regions of liquid oil in crystals of solid fats. For temperatures near the end of the metastable regime, the system will likely comprise crystals of solid fats in a sea of liquid oil. If the temperature is now rapidly decreased to $T<T^{*}$, the solid fat crystals can act as nucleation centres for the formation of crystal growth and the system will seemingly have exhibited “crystal memory” of the solid fats. We propose that this type of “crystal memory” arises because of the co-existence of the high temperature phase and the low temperature phase when the system is superheated into the metastable regime at $T>T^{*}$. This coexistence has been reported by Sadeghpour et al. [16].

The effect of “crystal memory” of the solid fat phase can be eliminated by holding the temperature sufficiently greater than $T^{*}$ for a sufficient length of time to let the system relax to the equilibrium state (upper solid curve for $T>T^{*}$). This is the process of “erasing crystal memory”. The temperature at which this takes place is not unique and a distribution of pairs of holding temperature-time, determined by the random processes which drive the relaxation to the equilibrium state, will be manifested. An analogous distribution of temperatures at which the metastable liquid state relaxes to the equilibrium state (lower solid curve for $T<T^{*}$), will be observed.

### 2.5. Predictions of the Mean Field Approximation (MFA)

It is known that, for a system undergoing a continuous phase transition, an MFA gets wrong the value of *T_c_*. However, it is useful to use the MFA since it can enable us to answer the following question: by changing parameters experimentally, are we able to drive an edible fats system to the critical point and thus locate it? The advantage of this is that we might then tune a system to occupy some pre-selected states. To make our point, we restrict the interactions between the model TAG molecules to nearest neighbours and simplify the parameters by choosing $E_{g}=0$, $D_{g}=1$, which merely defines the zero of energy and assumes that the state *g* is unique, and $J_{ge}=J_{ee}=0$, which are realistic assumptions, then $J=J_{gg}/4$ and $H\left(T\right)=J+E_{e}-k_{B}T\ln D_{e}$. If we ignore the term in $H\left(T\right)$, which yields the result of Figure 2A, we find the well-known MFA solution:

(6) $$\langle \sigma \rangle =\tanh\left[Jz\langle \sigma \rangle /k_{B}T\right]$$

where *z* = the average number of nearest neighbour hydrocarbon chains. This yields $T_{c}=Jz/k_{B}$. By including the temperature-dependent “field” term, $H\left(T\right)$, Equation (4) gives us:

(7) $$T^{*}=\frac{J+E_{e}}{k_{B}\ln D_{e}}$$

Salem [40] showed that, for two parallel identical *all-trans* hydrocarbon chains of length L, the dispersion component of J is proportional to L. Pink et al. [41] and Caillé et al. [42] argued that, to first order, $E_{e}=E_{0}L$ and $D_{e}=D_{e0}3^{L}$. The factor $D_{e0}$ must be a function of *L*, for, if the chain length is zero, then there is only one state possible. Accordingly, we can write $D_{e0}\left(L\right)=1\;\left(L=0\right),\;\;D_{e0}\left(L\right)=D_{0}\;\left(L>0\right)$. We should also take into account the electrostatic interactions (Keesom and Debye interactions) between the glycerol moieties, and we call this interaction $J_{C}$ [43,44]. These interactions come about because the glycerol core possesses a distribution of partial charges which exist due to the relative electronegativity of the atoms in the core. Pink et al. [41] and Caillé et al. [42] were not concerned with the degeneracy of the glycerol core. Here, we should include it as a multiplicative factor in the excited state degeneracy. If we write $J=J_{C}+J_{0}L$ and assume that the glycerol core possesses a degeneracy $D_{C}$, independent of *L*, then we obtain:

(8) $$T_{c}=\frac{z}{k_{B}}\left(J_{C}+J_{0}L\right)$$

(9) $$T^{*}=\frac{\left(\left(z/2\right)\left(J_{C}+J_{0}L\right)+E_{0}L\right)}{k_{B}\left(L\ln3+\ln\left(D_{C}D_{e0}\left(L\right)\right)\right)}$$

The ratio, $T_{c}/T^{*}$ is:

(10) $$\frac{T_{c}}{T^{*}}=\frac{z\left(J_{C}+J_{0}L\right)\left(L\ln3+\ln\left(D_{C}D_{e0}\left(L\right)\right)\right)}{\left(z/2\right)\left(J_{C}+J_{0}L\right)+E_{0}L}$$

We can consider two limits: L→∞ and L→0. Although the second limit is contradictory since the model is concerned with long-range order brought about by interacting chains and, with L=0, there are no chains, it is nonetheless, useful to explore since it approximately represents short chains. To consider this second limit, we set the degeneracy of the pure glycerol core to be $D_{C}=1$. We then obtain $\;\underset{L\to 0}{\lim}\left(T_{c}/T^{*}\right)=0$.

(11) $$\underset{L\to 0}{\lim}\left(T_{c}/T^{*}\right)=0$$

(12) $$\underset{L\to \infty }{\lim}\left(T_{c}/T^{*}\right)=2zJ_{0}L\ln3/\left(zJ_{0}+2E_{0}\right)$$

Equation (11) says that, if, for a given value of *L*, $T_{c}>T^{*}$ so that a discontinuous transition is manifested (Figure 2B), then, as *L* decreases, $T_{c}$ will decrease until $T_{c}<T^{*}$. Equation (12) says that, as *L* gets sufficiently large, $T_{c}/T^{*}$ is proportional to *L* and is thus unbounded.

### 2.6. Lifetimes of Metastable States

**Figure 4:**
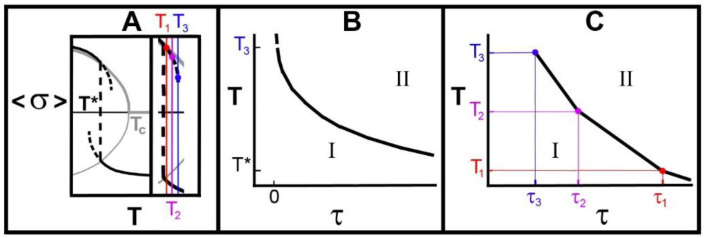
Schematic relationship between the metastable regime of the g-e model and time-temperature data. (**A**) Discontinuous transition of the g-e model and the metastable region (from Figure 2B) showing the transition region enlarged. Also shown are three isothermal processes occurring at equally-spaced temperatures $T_{1}<T_{2}<T_{3}$. (**B**) Schematic solution to Equation (14) (solid line) showing the lifetime, τ, of the superheated state at temperature, *T*. The dash at $T_{3}$ indicates that the system is no longer in the superheated regime. In region I, the system will return to the crystalline state from which it melted if cooled. In region II, the system will not return to the crystalline state when cooled (**C**) Plot of lifetimes-temperature relationship for $\tau_{3}<\tau_{2}<\tau_{1}$ using Equation (13).

We reiterate that we have modelled a system exhibiting a single discontinuous transition. We now relate the metastable regime associated with the discontinuous phase transition (Figure 2B) of the g-e model, the binodal (coexistence curve), to the relaxation time-temperature diagram obtained from a consideration of isothermal relaxation. Figure 4A shows a schematic cartoon of the discontinuous transition together with an expanded view of the metastable regime, the binodal (dashed curves), and three equally-spaced temperatures, $T_{1}<T_{2}<T_{3}$. The first is very close to the transition temperature, $T^{*}$, while the third is very close to the end of the metastable regime. We assume that the lifetime of the superheated metastable state at temperature, T, follows a modified Vogel-Fulcher-Tammann (VFT) equation [45,46,47,48,49]:

(13) $$\tau \left(T,\;T^{*}\right)+\tau_{0}=\tau_{0}exp\left[D^{*}T^{*}/\left(T-T^{*}\right)\right]$$

where $T\geq T^{*}$, $D^{*}$ is a strength parameter, related to the energy barrier between the metastable state and the equilibrium melted state, and $\tau_{0}$ is a pre-factor. In what follows we write $\tau_{n}=\tau \left(T_{n},\;T^{*}\right)$, and rewrite Equation (13) as:

(14) $$\ln\left(1+\;\left(\tau_{n}/\tau_{0}\right)\right)=D^{*}T^{*}/\left(T_{n}-T^{*}\right)$$

Ignoring that this equation is applicable only in the superheated metastable regime, note that as $T_{n}\to T^{*}$, τ→∞, a stable state, and that as $T_{n}\to \infty$, τ→0. By calculating $\partial \tau_{n}/\partial T_{n}$ it is trivial to show that the slope of (13) is monotonic negative as $T_{n}$ increases, and changes from −∞ when $T_{n}\to T^{*}$, to 0 as $T_{n}\to \infty$, as shown in Figure 4B. We also calculated $\tau_{n}$ explicitly for three temperatures. It should also be noted that it is possible that, as $T_{n}\to T^{*}$, $D^{*}\to 0$ so that $\tau_{n}\to 0$.The temperatures, $T_{n}$, were chosen to be equally spaced and the approximate values of the average lifetimes, $\tau_{3}<\tau_{2}<\tau_{1}$, were identified from Equation (13). Finally, we have plotted these lifetimes versus their temperatures and this is shown in Figure 4C. It should be noted that, because $\tau_{3}<\tau_{2}<\tau_{1}$ and the temperatures are equally spaced, the curve is confirmed to be convex as shown in Figure 4B.

**Figure 5:**
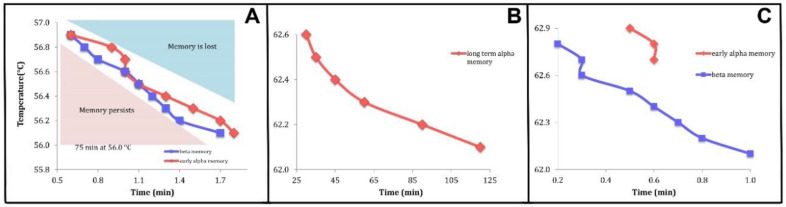
Time-temperature relationships [1]. (**A**): Trimyristin (MMM). Short-time time-temperature boundaries of two types of memory. The red points are for early α memory, and the blue points represent the temperature and time combination of β memory. (**B**): Tripalmitin (PPP). Long term α memory. (**C**): Short-time time-temperature boundaries for early α memory (red) and β memory (blue). Used with the kind permission of Yujing Wang.

**Figure 6:**
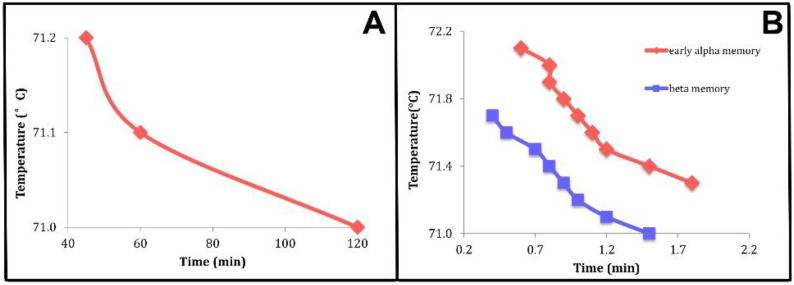
Time-temperature relationships for tristearin (SSS) [1]. (**A**) Long-time time-temperature boundaries. (**B**) Short-time time-temperature boundaries for early α memory (red) and β memory (blue). Used with the kind permission of Yujing Wang.

Also shown are regions I and II. Region I is characterized by the ability of the system to return to the crystalline state from which it melted if it is cooled. Our assertion is that it is this that is “crystal memory”. In region II, the system will not return to the crystalline state when cooled. We have found that some of the results of time-temperature studies [1] are in accord with the prediction of Figure 4B,C. These are shown in Figure 5 and Figure 6.

### 2.7. Results from Theoretical Model, Crystal Memory, Metastable Regimes, and Relaxation

**Figure 7:**
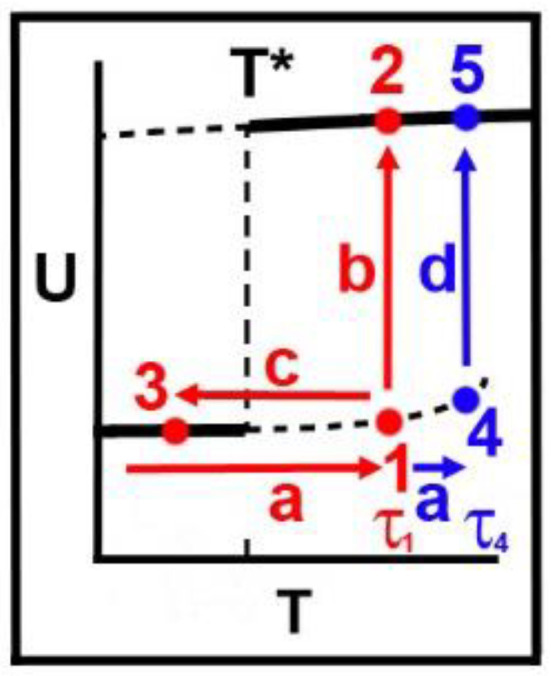
Enthalpy, U, of the model (Figure 3B) as a function of temperature, T, showing the phase transition at temperature $T^{*}$. Heavy solid lines indicate equilibrium enthalpies in a mean field approximation. Short-dashed lines indicate metastable regimes. Lifetimes, below which memory persists and above which memory is lost (Figure 4C) are indicated by $\tau_{1}$ and $\tau_{4}$. We predict that $\tau_{4}<\tau_{1}$.

We can now relate the metastable regimes of the model to understand our proposal for the physical basis of the crystal memory effect shown in Figure 4, and this is shown in Figure 7. There, a portion of the enthalpy versus temperature curve (Figure 3B), including the discontinuous transition at $T=T^{*}$, is shown. Also shown are two temperature ramps (a) to states 1 and 4 and two relaxations (b,d) to equilibrium states 2 and 5. Alternatively, the system can be cooled (c) from 1 to 3 thereby exhibiting a memory effect. A similar procedure (not shown) would involve cooling from state 4. The times $\tau_{1}$ and $\tau_{4}$ represent the average lifetimes that one can hold the system in the metastable regimes 1 and 4 before they relax to the equilibrium states 2 and 5, respectively.

In order to eliminate crystal memory, one must wait for the system to relax. Thus, for example, heat transfer, or energy transfer via mixing which could involve shearing the sample, should be considered as possible relaxation protocols.

It is well-known, and in accord with Equation (13), that this simple system will relax, on the average, more quickly from the superheated metastable regime to the equilibrium high-temperature phase, the higher is the temperature. Accordingly, we will find that $\tau_{4}<\tau_{1}$ as shown for the lifetimes in Figure 4.

## 3. Discussion

### 3.1. Predictions to Test the Model

From Equations (11) and (12) we saw that if, for a given value of chain length *L*, $T_{c}>T^{*}$ so that a discontinuous transition is manifested (Figure 2B), then, as *L* decreased, $T_{c}$ will decrease until $T_{c}<T^{*}$. In the limit of very long chain length, Equation (12) says that, as *L* gets sufficiently large, $T_{c}/T^{*}$ is proportional to *L* and is thus unbounded. This enables us to provide a test of the model.

We predict that the first limit might be manifested by studying the phase transitions of solid saturated TAGs with progressively shorter hydrocarbon chain lengths, *L*. As long as crystals are formed, fluctuations will become larger as the chain length shortens. Eventually, the chain length might be sufficiently short that crystalline state long-range order will still be manifested and the critical temperature, $T_{c}$, will be reached ($T^{*}=T_{C}$). This should be manifested by large fluctuations at the phase transition. As *L* is further decreased and if crystalline order still occurs, then a broad transition (Figure 2C) will be observed with fluctuations smaller than those when $T^{*}=T_{C}$. Conversely, as *L* is increased, $T_{C}$ moves to higher temperatures and the temperature gap between it and $T^{*}$ becomes larger so that the transition enthalpy increases as the discontinuity (Figure 2B and Figure 3B) becomes larger.

It is also predicted that, in our model, “crystal memory” will be absent as the hydrocarbon chains get shorter with $T^{*}\geq T_{C}$. This is because there is no longer a discontinuous phase transition and therefore, in this model, no metastable regions.

### 3.2. Relating Experimental Data to Theoretical Calculations. Crystal Memory. Holding Time-Temperature Relationship

We have identified the theoretical metastable state lifetimes with the holding times defining the boundaries separating the regions in which crystal memory is, or is not, observed: regions I and II of Figure 4B,C, Figure 5 and Figure 6 below where those regions are labelled “memory persists” and “memory is lost”, respectively.

Wang [1] carried out time–temperature studies of trimyristin (MMM), tripalmitin (PPP), and tristearin (SSS), shown in her thesis as Figures 4.18, 4.24, and 4.29, respectively, and adapted for this paper as Figure 5 and Figure 6, with her kind permission.

Two types of memory were identified in pure SSS [1]: “β memory” (BM) and “early α memory” (EAM). The “early α memory” comprised a short term (SEAM) and long term (LEAM) memory. EAM was reported as being not related to epitaxial growth on an existing crystal matrix.

Time-temperature boundaries of each one of these memories were characterized by exploring many holding temperature-time combination pairs.

BM was considered persistent when the recrystallization cooling ramp [1] resulted in the direct formation of the β polymorph, i.e., without crystallization of the α or β’ polymorph. It occurred at temperatures higher than the initial crystallization of the α polymorph during a cooling ramp. EAM was considered persistent when a recrystallization cooling step resulted in the formation of the α polymorph at a temperature higher than its formation during a ramp.

Our model assumes only a single discontinuous phase transition so that it will not reproduce the complexities of Wang’s data. However, we can see that the general dependence of the holding time-temperature curves exhibit the same dependence as do the curves defining the relaxation times from the superheated metastable state to the equilibrium state at a given temperature.

Two things can be seen in Figure 5 and Figure 6: (i) the relationships have the general appearance of the curves shown in Figure 4B,C and (ii) although the temperatures shown concerning “α memory” are greater than the phase transition temperatures of the α phase of these systems, the temperatures shown that involve “β memory” are less than those listed for the β phase transition temperatures of MMM [50], PPP [51], and SSS [52].

## 4. Conclusions

We have investigated the physical basis of “crystal memory” effects by making a toy model (g-e model) exhibiting a single discontinuous (i.e., first-order) phase transition which incorporates what we know about the properties of hydrocarbon chain states and their interactions. Discontinuous phase transitions can exhibit heating and cooling metastable regimes and it is these regions that we have identified as being the physical basis of “crystal memory” at triacylglycerol phase transitions. The thrust of this paper is to propose that the existence of “crystal memory” is attributable to the existence of such metastable regimes.

The model represented each TAG molecule by two states as used previously [26] which interact via short range interactions. Here, we distinguished between the hydrocarbon chain interactions and the glycerol core interactions. However, the model does not encompass polymorphic states, and these might turn out to be essential.

The protocol used to investigate and characterize the “crystal memory” effect involved a tempering stage followed by a testing stage [1]. This was defined by temperature ramps, comprising heating or cooling ramps of 15 °C/min or 20 °C/min with isothermal plateaus separating the ramps. Finally, the system was kept at a holding temperature to search for a memory effect.

In order to relate metastable state lifetimes to temperature, we proposed an extension of the Vogel-Fulcher-Tammann equation to superheated regions, shown in Equation (13).

With a transition temperature defined as $T=T^{*}$, we have shown that:

1. There is a critical point at $T=T_{c}$ which plays an important role in whether there is a discontinuous transition, a continuous transition or a crossover with no divergences in thermodynamic quantities, at $T=T^{*}$.
2. The dependence of $T_{c}$ and $T^{*}$ upon hydrocarbon chain length, *L*, are different. The effect of this is that, as *L* goes to zero, $T_{c}/T^{*}$ goes to zero, while, as *L* gets sufficiently large, then $T_{c}/T^{*}$ depends linearly upon *L* and so becomes large. By changing the hydrocarbon chain length, *L*, this offers a way to observe changes in the phase transition or crossover, as shown in Figure 2.
3. Measurements of the average holding times, below which “crystal memory” is exhibited and above which all such memory is lost [1], showed that this time has an inverse relationship to the temperature as predicted by the g-e model and shown in Figure 4B,C.
4. We showed that the observed “crystal memory” effect could be explained by the existence of the metastable regimes (Figure 4 and Figure 7). The model says that the average time, the holding time, to relax to the high-temperature equilibrium state must decrease, the higher the temperature, as reported in point 3.

Although this model does not consider the existence of the three solid phases, α, β’, and β, this omission is not immediately relevant to the message proposed here: that the “crystal memory” effect is due to the system accessing metastable regimes which are a consequence of the discontinuous nature of the phase transition at $T^{*}$. Although many studies have been carried out on these three phases, there exists no mathematical model which describes all three phases in a unified way, based upon molecular interactions, and which accounts for their phase transitions. Future work will be concerned with including those three phases.
